# Radiofrequency Ablation of Leiomyomas via a Single-Site Incision

**DOI:** 10.7759/cureus.81558

**Published:** 2025-04-01

**Authors:** Ovgu Barut, Jose Luis Terrazas

**Affiliations:** 1 Obstetrics and Gynecology, Health Corporation of America Florida Northwest Hospital, Margate, USA

**Keywords:** gynecologic surgery, laparoscopic surgery, leiomyomas, obgyn, single site surgery

## Abstract

Laparoscopic radiofrequency ablation (RFA) is a minimally-invasive technique to decrease the size of tumors using thermal energy to destroy tissue. Uterine leiomyomas are common conditions comprising benign smooth muscle tumors. Single-incision laparoscopic surgery is a modern surgical approach for allowing the removal of diseased organs through a small skin incision. We aim to demonstrate the feasibility and advantages of a single-site RFA for uterine leiomyomas, highlighting its potential to reduce operative time, minimize scarring, and improve patient recovery compared to traditional multi-port approaches. We present the case of a 46-year-old female patient who presented for scheduled RFA of a dominant 4.5 cm anterior myoma causing heavy menstrual bleeding. A uterine manipulator was introduced into the endometrial cavity. A single-site port was introduced into the abdomen and laparoscopic ultrasound was then performed revealing a 4.5 cm anterior myoma as well as a posterior myoma The anterior aspect of the fibroid was desiccated using an RFA probe set at 95°C for two minutes. The probe was then repositioned to the posterior aspect and desiccated in multiple locations until complete treatment was achieved. This case demonstrates the successful treatment of a 4.5 cm anterior uterine myoma and posterior fibroid using single-site RFA, resulting in minimal blood loss, reduced postoperative pain, and rapid recovery. The surgery was performed in a manner identical to traditional laparoscopic RFA with three ports. In addition to minimal blood loss, our single-site approach resulted in less incisional pain, less scarring, and better cosmesis. The patient was discharged on the same day and rapidly resumed her activities of daily living as assessed during her postoperative visit. Our case highlights the benefits of novelty in medicine with increased patient satisfaction regarding cosmesis. This technique has the potential to reduce costs by reducing operative time and facilitating a faster return to work for the patient. With appropriately selected patients, similar access to most myomas can be achieved.

## Introduction

The use of radiofrequency energy to increase the temperature of tissues via coagulative necrosis was first described in 1891 by d’Arsonval [1]. Laparoscopic radiofrequency ablation (also known as Lap RFA) is a minimally-invasive technique that decreases the size of tumors, nodules, or any other abnormal growths in the body and uses thermal energy to destroy tissue [2]. It was first described in the treatment of uterine leiomyomas in 2002 [3]. Uterine leiomyomas are a common condition, comprised of benign smooth muscle tumors [4]. They occur in 70% of Caucasian women and 80% of African-American women by the age of 50. They are the leading cause of hysterectomy in the United States and their economic impact is considerable, costing about 34 billion dollars annually [4,5].

Laparoendoscopic single-site (LESS) surgery (also known as single-incision laparoscopic surgery) is a modern surgical approach for the removal of diseased organs through a small incision on the skin. While 12.3 million US women between the ages of 25 and 44 suffer from symptoms of fibroids, the number of procedures done is less than 2,50,000 annually due to their invasive nature. This led to the invention of RFA for fibroids. With this technique, all the conventional laparoscopic ports are condensed to one surgical incision and the surgery is done via a single entry point, usually through the navel [6]. Single-port laparoscopy has been successfully applied in various gynecologic procedures, demonstrating comparable surgical outcomes to traditional laparoscopy while minimizing scarring and improving patient satisfaction [7].

## Case presentation

A 46-year-old female patient, gravida 3 para 3003 (i.e., with three living children) with a past medical history of abnormal uterine bleeding due to leiomyomas presented for scheduled RFA of uterine myomas. The patient’s surgical history was significant for bilateral tubal ligation in 2009. She had no other past medical history, had been experiencing abnormal uterine bleeding for two years, and desired definitive management. During preoperative imaging, a pelvic ultrasound showed a dominant 4.5 cm myoma in the anterior portion of the uterus. 

**Figure 1:**
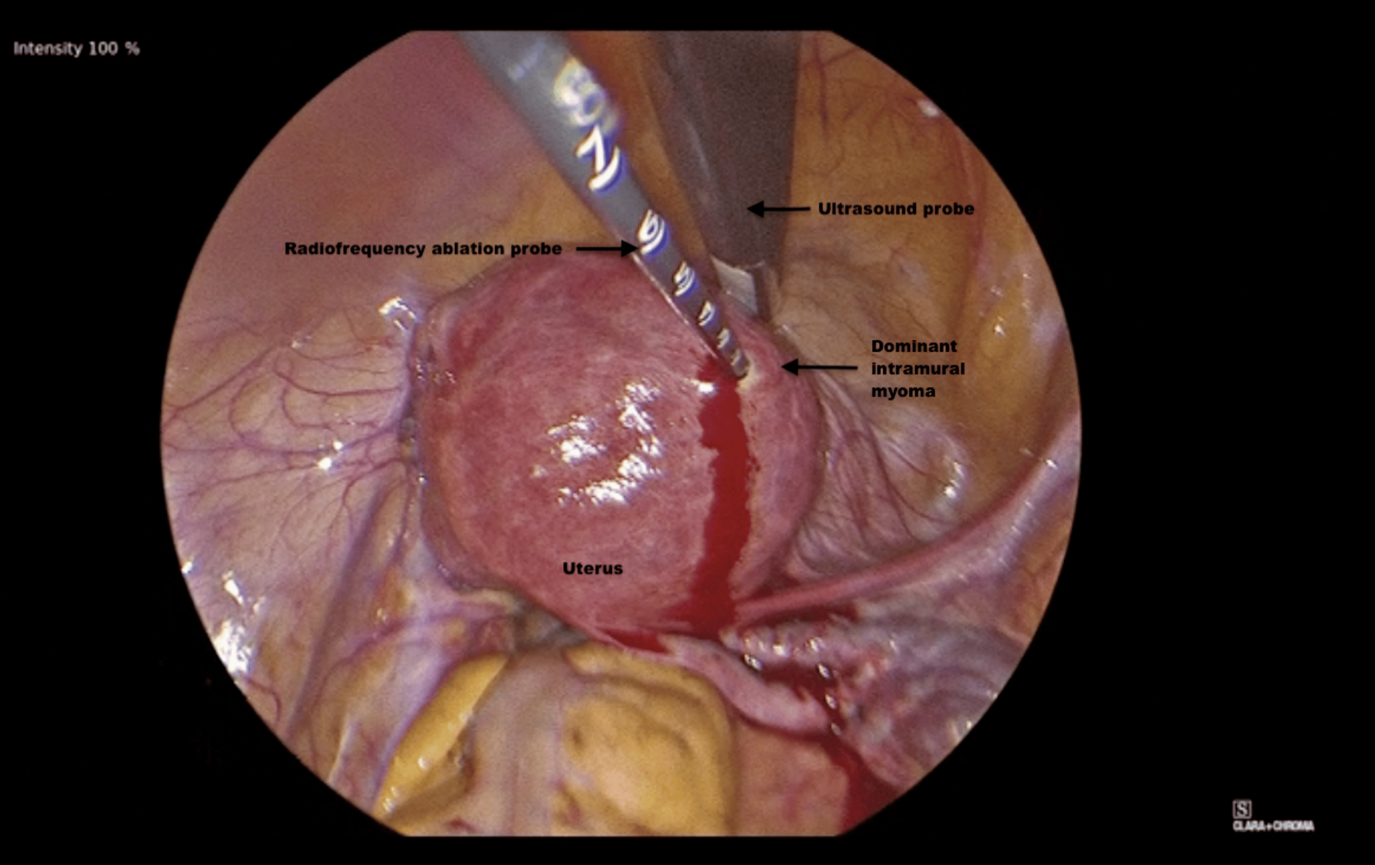
The anterior fibroid undergoing radiofrequency ablation

During the procedure, a uterine manipulator (VCare DX, Conmed Corporation, Florida, US) was introduced into the endometrial cavity. A single site port was introduced into the abdomen and laparoscopic ultrasound was then performed. It revealed an anterior myoma measuring approximately 4.5 cm. The anterior aspect of the fibroid was then desiccated with the RFA probe (Acessa Model 7100, Acessa Health Inc, Texas, US) for 2 minutes (Figure 1). 

**Figure 2:**
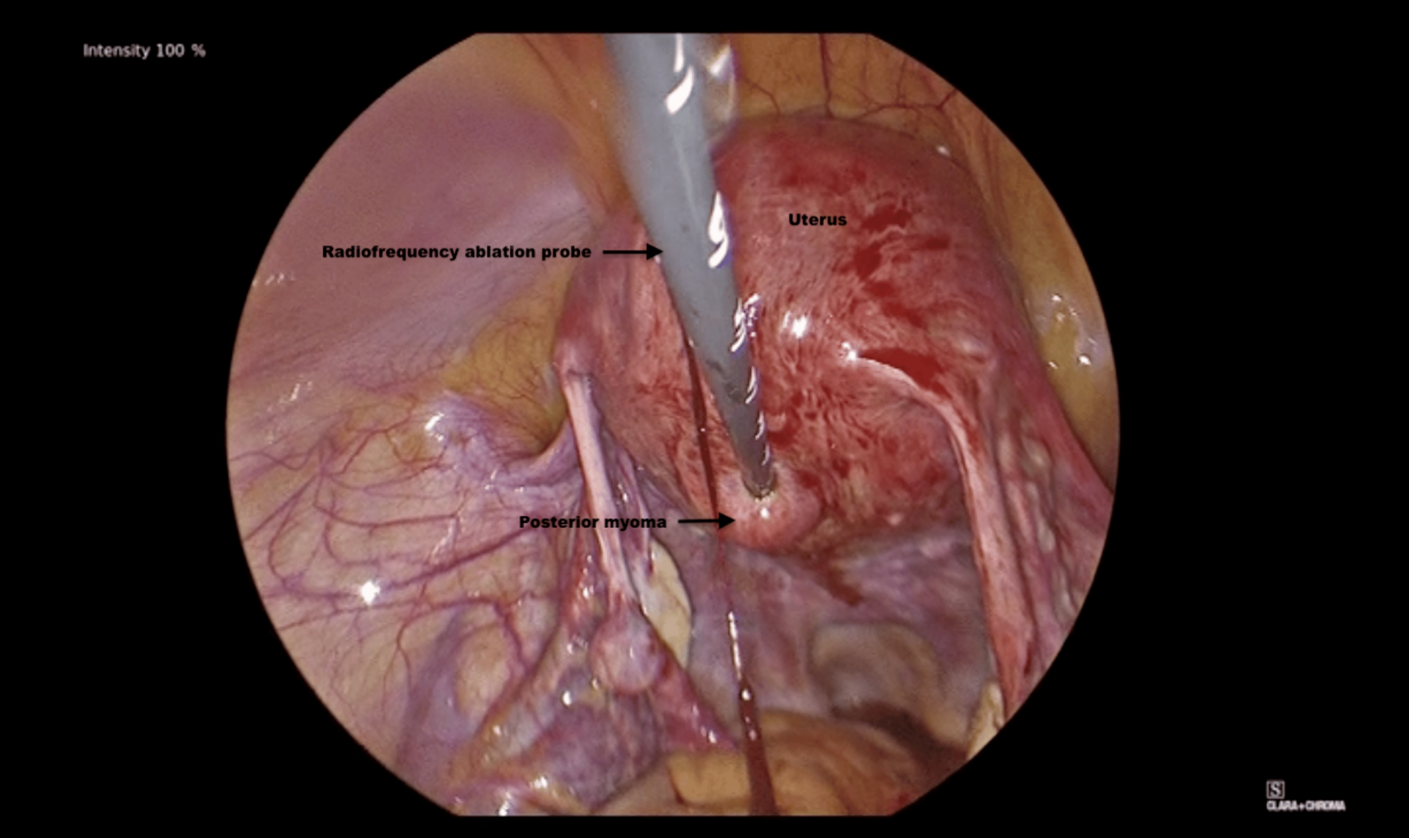
The posterior fibroid undergoing radiofrequency ablation

A posterior fibroid was also identified at the time of the procedure. The probe was then repositioned to the posterior aspect and desiccated in multiple locations until completely treated (Figure 2). 

The defect created by the probe was cauterized with monopolar cautery. The port was then removed. 

The fascia was reapproximated using 1 Stratafix suture (Ethicon Inc., New Jersey, US) in a running fashion. The skin was reapproximated in the usual fashion. The estimated blood loss was about five milliliters. There were no intraoperative or postoperative complications. The patient’s postoperative course was uneventful and she was discharged on the same day. The patient was followed up in the office two weeks after the operation and the incision sites had healed appropriately. Follow up imaging was not indicated during the postoperative course. 

## Discussion

Uterine leiomyomas, also known as fibroids, fibromyomas, fibromas, and myomas, are defined as smooth cell tumors. They are benign growths and arise from the myometrium. Fibroids may be found in up to 70% of women and are largely found in premenopausal women. Leiomyomas are five times more common in Black women than in White ones [4]. The classification of fibroids is determined by their location in the uterus. Subserosal fibroids are found under the outer layer of the uterus, intramural ones within the uterine wall, whereas submucosal fibroids are located within the uterine cavity [5]. Depending on their size and location, they may cause heavy and prolonged uterine bleeding.

There are various treatments available for uterine fibroids and these can be divided into medical and surgical ones. Medical treatments are divided into non-hormonal and hormonal therapies. The non-hormonal options include non-steroidal anti-inflammatory drugs (NSAIDs) and tranexamic acid. Hormonal treatments include oral contraceptive pills (OCPs), levonorgestrel intrauterine systems, gonadotropin-releasing hormone agonists, progesterone receptor modulators, or aromatase inhibitors. NSAIDs are the first-line treatment for patients who consider non-hormonal options. Tranexamic acid, an antifibrinolytic medication, may also be considered to treat heavy bleeding that is associated with uterine leiomyomas. A major side effect of tranexamic acid is increased risk of deep vein thrombosis and pulmonary embolism. Medical treatments to help control fibroid growth include gonadotropin-releasing hormone agonists. These agonists cause a menopause-like state by inducing low estrogen which reduces the size of the tumor and shrinks the size of the uterus. This also induces the beneficial side effect of amenorrhea which may be helpful in women with iron deficiency anemia due to the chronic blood loss from abnormal uterine bleeding. However, these medications may not be considered for long-term use as the menopausal state predisposes women to decreased bone density and fractures. Other medical modalities that may be considered but do not decrease the size of the fibroid include progestins (such as medroxyprogesterone acetate and norethindrone), as well as the Mirena IUD (also a progestin). These are synthetic forms of progesterone used to decrease bleeding. Some common side effects include bloating, headache, nausea, fatigue, and breast tenderness or pain. OCPs used to regulate and decrease blood flow during menses have a similar side effect profile as the progestins, including nausea, breast tenderness, headache, and bloating. 

Surgical options for treating uterine fibroids include hysterectomy or removing the uterus, which is the definitive management of the condition as it prevents recurrence. There are different gynecologic approaches to hysterectomy including abdominal, vaginal, laparoscopic, or robotic options. The approach depends on the patient's medical history, uterine size, and the skill set of the surgeon [5]. This option is limited only to women who do not desire further childbearing. Another surgical option for the removal of fibroids is known as myomectomy. This is often done when fertility is desired. Myomectomies can also be performed through the abdominal, vaginal, laparoscopic, and robotic approaches, much like hysterectomies. Depending on the location of the fibroid, a hysteroscopic approach can also be taken. Hemorrhage remains the most important concern during myomectomies and are mainly associated with the abdominal approach [5,8,9]. RFA guided by laparoscopic ultrasound is a minimally-invasive technique that has gained traction in the treatment of uterine fibroids due to its ability to achieve volumetric ablation with minimal damage to the surrounding tissues. 

Procedural interventions to treat fibroids also include uterine artery embolization (UAE), high-intensity focused ultrasound, magnetic resonance high-intensity focused ultrasound. For patients who do not desire to undergo surgery, this method may be considered. UAE involves placing a catheter through an incision in the groin and guiding it to the uterine arteries. This catheter is then used to block off blood vessels that feed the uterine leiomyomas [10]. Although this procedure does not excise the fibroids, it may help to decrease their size by up to 50% [10]. For appropriate candidates, this procedure offers rapid recovery without the need for further medical therapy. Though it results in a shorter hospital stay and less blood loss compared to hysterectomy or myomectomy, the patient's quality of life is still compromised.

RFA became widely used in modern medicine upon the invention of the Bovie knife in 1828, used for cauterization and cutting of tissue using radiofrequency current [2]. It is used to treat a wide range of conditions including benign tumors in different body systems as well as chronic pain [1]. The electrode of the RFA functions as the cathode of an electric circuit. This circuit is closed by the dispersing pads applied to the patient’s thighs [8]. Once the circuit is turned on, tissue damage occurs only to the area around the electrode.

RFAs are increasingly utilized as a uterine sparing, minimally-invasive method of treating uterine myomas. The patient is preferentially placed in the dorsal position with a single-tooth tenaculum applied to the cervix to manipulate and stabilize the uterus. Typically, RFA of fibroids are done while using three ports; a 5 mm port for the laparoscope and a second 10-12 mm port for the laparoscope. A rigid ultrasound transducer is used to verify the appropriate placement of the device within each myoma as well as the third port, which is the transabdominal percutaneous handpiece [9]. During the RFA of myomas, minimal damage is done to the myometrium and serosa due to volumetric ablation of the leiomyoma. This reduces the common symptoms of leiomyomas such as abnormal uterine bleeding and symptoms caused due to its bulk such as abdominal pain and constipation. RFA offers a minimally- invasive alternative to traditional open surgical approaches for the treatment of uterine leiomyomas. While open surgeries, such as abdominal myomectomy or hysterectomy, are effective, they are associated with less common but serious complications, including postoperative adhesions, hemorrhage, and the risk of retained surgical foreign bodies (gossypiboma), as highlighted in recent case reports [11]. In contrast, RFA minimizes these risks by utilizing thermal energy to achieve targeted fibroid ablation with minimal damage to the surrounding tissues. This approach is associated with significantly reduced intraoperative blood loss compared to open or traditional laparoscopic myomectomy [12-14], while reducing postoperative pain, facilitating faster recovery, and improving cosmesis. These advantages make RFA a preferable option for selected patients. 

We present a case of single-site RFA where multiple uterine myomas were effectively treated with this minimally-invasive technique with a short recovery time and rapid effect on adverse symptoms. The surgery was performed in the same manner as traditional laparoscopic RFA with three ports. With appropriate patient selection, the same access to most myomas can be achieved. In addition to minimal blood loss during surgery, our single-site approach led to less incisional pain, less scarring, and better cosmesis.

Our patient was discharged on the same day of surgery. She was, therefore, able to resume her activities of daily living rapidly. This case represents an innovative and creative approach using what is already commercially available. In addition to its clinical efficacy, our case demonstrates that single-site RFA offers improved cosmesis and patient satisfaction compared to traditional multi-port approaches. This technique has the potential to reduce cost by decreasing operative time and facilitating a faster return to the workforce for the patient. Moreover, our case emphasizes inventiveness in minimally-invasive gynecologic surgery by creatively using the single-site approach compared to the traditional option.

## Conclusions

Minimally-invasive surgery has increased in popularity and is a standard choice of surgery for most obstetricians and gynecologists. Myomectomies are a common type of surgery performed in this field and are associated with greater blood loss and postoperative complications compared to other major gynecologic surgeries such as hysterectomies. RFA is a newer technique that treats abnormal uterine bleeding due to leiomyomas and may be performed laparoscopically with better cosmesis. This type of surgery, in addition to decreased blood loss, promotes quick recovery and decreases operative time. Moreover, we advocate for the creative approach of single-site RFA in appropriately selected patients, as it offers reduced operative time, improved cosmesis, and faster recovery compared to traditional techniques. Further studies are needed to evaluate long-term outcomes and broader applicability of this approach.
